# Towards an adiposity-related disease framework for the diagnosis and management of obesities

**DOI:** 10.1007/s11154-023-09797-2

**Published:** 2023-05-10

**Authors:** Carolina M. Perdomo, Icíar Avilés-Olmos, Dror Dicker, Gema Frühbeck

**Affiliations:** 1 Department of Endocrinology and Nutrition. Clínica, Universidad de Navarra, Pamplona, Spain; 2 IdiSNA (Instituto de Investigación en la Salud de Navarra), Pamplona, Spain; 3 CIBEROBN, Instituto de Salud Carlos III, Madrid, Spain; 4 Department of Neurology, Clínica Universidad de Navarra, Pamplona, Spain; 5 Department of Internal Medicine D, Rabin Medical Center, Hasharon Hospital, Petah Tikva, Israel; 6 Sackler School of Medicine, Tel Aviv University, Tel-Aviv, Israel

**Keywords:** Obesity, Visceral adipose tissue, Cardiovascular disease, Adiposity-based chronic disease, Obesity-related adipose tissue disease, Adiposopathy

## Abstract

Obesity is a complex disease that relapses frequently and associates with multiple complications that comprise a worldwide health priority because of its rising prevalence and association with numerous complications, including metabolic disorders, mechanic pathologies, and cancer, among others. Noteworthy, excess adiposity is accompanied by chronic inflammation, oxidative stress, insulin resistance, and subsequent organ dysfunction. This dysfunctional adipose tissue is initially stored in the visceral depot, overflowing subsequently to produce lipotoxicity in ectopic depots like liver, heart, muscle, and pancreas, among others. People living with obesity need a diagnostic approach that considers an exhaustive pathophysiology and complications assessment. Thus, it is essential to warrant a holistic diagnosis and management that guarantees an adequate health status, and quality of life. The present review summarizes the different complications associated with obesity, at the same time, we aim to fostering a novel framework that enhances a patient-centered approach to obesity management in the precision medicine era.

## Introduction

In the last years, efforts have focused on addressing obesity beyond a body mass index (BMI) perspective, since dysfunctional adiposity promotes several diseases [1]. People living with obesity (PlwO), have a higher risk mortality from all causes, with cardiovascular disease (CVD) together with cancer as standing out [2, 3]. The adiposity-based chronic disease (ABCD) concept has been proposed to improve the diagnosis of obesity based on the dimensions of etiology, severity of adiposity excess, and assessment of health risks [1]. This novel diagnostic framework aims to promote an accurate comorbidity screening in a systematic manner leading to an enriched patient care. Recently, the term “obesity-related adipose tissue disease” (OrAD) has been proposed to collectively englobe the diverse pathologies related to “adiposopathy”, which include hypertrophy, inflammation and fibrosis of the adipose tissue (AT) [4]. The present review fosters a novel framework based on dysfunctional adiposity recommending a holistic view with a patient-centered approach in the precision medicine era.

### Common pathophysiology in obesity-related diseases

**Fig. 1:**
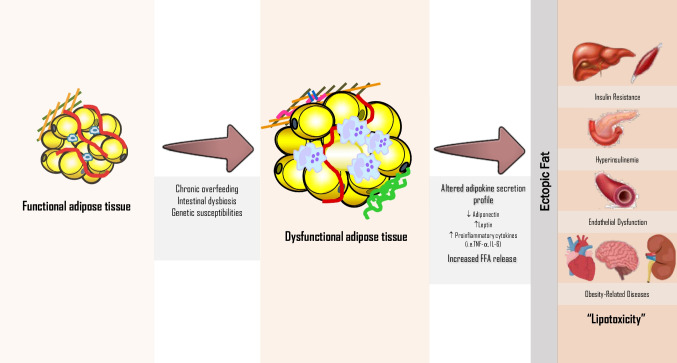
Dysfunctional adipose tissue enlargement underlies ectopic fat accumulation Diverse factors may influence the appearance of a dysfunctional adipose tissue, which through a continuum of altered adipokine secretion and increased FFA release promotes ectopic fat accumulation FFA: free fatty acids

Obesity-related diseases are predominantly determined by physical (i.e. hypoventilation, osteoarthritis) and metabolic features [1]. AT produces a variety of molecules called adipokines to maintain homeostasis (i.e. thermoregulation, energy storage, insulin sensitivity, and immunity, among others) [5]. AT dysfunction underlies the mechanisms linking obesity and the development of metabolic comorbidities [5–7]. AT dysfunction typically appears due to the pathological enlargement of fat mass (hypertrophy and hyperplasia of adipocytes) [7, 8], with subsequent hypoxia as blood supply becomes insufficient. The increased recruitment of macrophages, dendritic cells, and lymphocytes leads to an adiponectin expression downregulation, along with release of pro-inflammatory adipokines via metabolic signaling pathways activation [9, 10]. This increases oxidative stress, insulin resistance (IR), dyslipidemia and incites progressive accumulation of ectopic fat [11–13]. Ectopic fat intensifies the pro-inflammatory cytokine activity favoring the development of lipotoxicity via oxidative stress, activation of platelets, elevated renin–angiotensin–aldosterone system activity, cellular senescence, and dysfunction of the endothelium, eventually underlying obesity-related diseases [13–15]. The different phenotypes of obesity have inflammatory cytokines levels that reflect the dysfunctional AT continuum implicated in the systemic inflammation [16]. Figure 1 summarizes the common pathophysiology in obesity-related diseases. Current trends attempt to foster a more personalized diagnostic and treatment approach of obesity based on adiposopathy [17].

## Organ systems approach in relation to dysfunctional adiposity

**Fig. 2:**
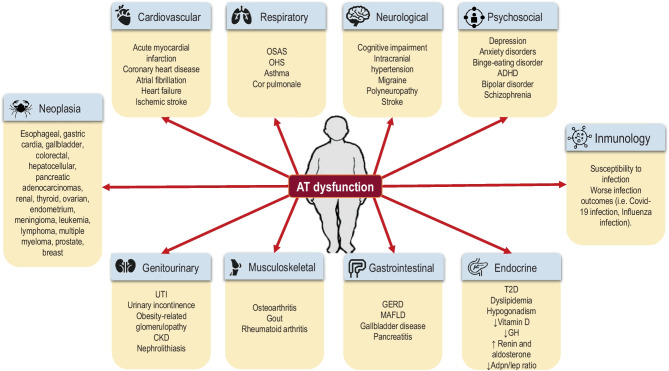
Obesity-related diseases. Obesity-related diseases derived from adipose tissue dysfunction ADHD: attention deficit hyperactivity disorder; Adpn/Lep: adiponectin/leptin; AT: adipose tissue; CKD: chronic kidney disease; GERD: gastroesophageal reflux disease; GH: growth hormone; MAFLD: metabolic associated dysfunction fatty liver disease; OSAS: obstructive sleep apnea syndrome; OHS: obesity hypoventilation syndrome; T2D: type 2 diabetes mellitus; UTI: urinary tract infection

Figure 2 summarizes the obesity-related diseases.

### Cardiovascular diseases

As a major independent ischemic heart disease risk factor, obesity also directly contributes to incident cardiometabolic risk like type 2 diabetes (T2D), dyslipidemia, sleep disorders, and hypertension [18]. Visceral adipose tissue (VAT) is frequently accompanied by collection of fat in physiologically lean tissues (liver, heart, skeletal muscle), which gradually promotes chronic inflammation, that enhances endothelial cell dysfunction and atherosclerosis, including acute thrombosis, associated with a higher CVD risk [19]. An international multicenter case–control study, involving more than 27,000 participants, reported the waist-height ratio (WHtR) as the strongest myocardial infarction predictor, independently of gender, age, smoking status, ethnicity, hypertension, diabetes, and dyslipidemia [20]. Regarding cardiac arrhythmias, obesity may account for one-fifth of the patients with atrial fibrillation [18]. Additionally, a dose–response meta-analysis and systematic review have evidenced that a BMI > 25 kg/m^2^ together with abdominal adiposity are related with an elevated heart failure risk [3]. The classical major adverse cardiovascular events (MACE) comprise nonfatal stroke, nonfatal myocardial infarction, and cardiovascular death. The elevated ischemic stroke risk is also related to obesity [21], as expected, although it seems to depend more on the metabolic consequences of obesity [22]. Interestingly, physical activity and weight loss attenuate the detrimental effects of obesity on CVD [23].

### Respiratory diseases

PlwO may have a mechanical compression of the chest cavity on the diaphragm, which may induce an increased pulmonary resistance, and reduced respiratory muscle strength, which may eventually lead to cor pulmonale [24]. Lung function and body fat distribution have a robust correlation, especially when fat accumulates in the thorax and in the abdomen [24]. In this line, asthma prevalence and severity are associated with excess total body weight, its incidence increases by 50% in patients with overweightness/obesity [25]. Likewise, the prevalence of pulmonary embolism is higher in PlwO than in people without overweightness [26]. Furthermore, overweightness is considered the most common risk factor of obstructive sleep apnea (OSA) [27]. OSA is traditionally related to an incremented cardiovascular risk and a reduced quality of life with mechanical and metabolic factors playing an important role in its etiology. In PlwO, OSA prevalence is estimated to be 19–31%. The coexistence of excess weight, daytime hypercapnia (pCO2 > 6 kPa) together with disrupted sleep breathing pattern characterizes the obesity hypoventilation syndrome (OHS) [24].

### Gastrointestinal diseases

The accumulation of intracellular fat in the liver characterizes metabolic associated fatty liver disease (MAFLD) [28]. Its prevalence worldwide is rising, especially in PlwO or T2D. A meta-analysis involving 8.5 million individuals reported that more than 80% of patients with fatty liver disease had overweightness, 72% had dyslipidemia, and 44% had T2D [29]. IR and visceral fat are the central mechanisms linking both entities [30]. There is evidence to consider MAFLD as an additional independent risk factor for CVD [31]. Moreover, MAFLD will become the first cause of liver transplantation [32]. The presence of fibrosis and its severity are the factors related to the increased all-cause mortality, however due to CVD mainly [33]. Obesity is also associated with esophageal disorders, specially gastroesophageal reflux disease (GERD) [34], which may lead to esophagitis, Barrett’s esophagus, or adenocarcinoma. Regarding other gastrointestinal disorders, PlwO have a higher risk for gallbladder disease [35]. Gallbladder dysmotility is the suggested mechanism to explain this association [36]. Likewise, an association between obesity and increased risk of acute pancreatitis has been reported [37].

### Endocrinological diseases

Obesity may have an impact on numerous endocrine organs, encompassing the hypothalamic-pituitary axis, vitamin D alterations and sex steroids disarrangements, among others [38]. IR is responsible for many endocrine abnormalities. In the presence of excess adiposity, increased plasma free fatty acids (FFA) concentrations are observed [30]. Mitochondrial fatty acid β-oxidation mediates lipid removal of the liver, subsequently, triacylglycerols reach the bloodstream as VLDL or can be accumulated as liver lipid droplets. When AT is overwhelmed with FFA, deposition of fat occurs in beta cells of the pancreas as well as in the liver and skeletal muscle [38]. Hepatic triacylglycerol deposition stimulates IR leading to a compensatory hyperinsulinemia that reduces the synthesis of glycogen, elevates uptake of liver FFA at the same time as inhibiting hepatic β-oxidation [15]. Moreover, hyperinsulinemia diminishes the hepatic hormone-binding proteins, frequently related to endocrine dysregulation [38]. All these alterations lead to an increased proinflammatory profile as described above.

The most common endocrinopathy in obesity is T2D [39]. Regarding other endocrinopathies [38], obesity is associated to hypogonadism through the reduction in the release of gonadotropin releasing hormone, the enhancement of aromatase (promoting free testosterone conversion to estrogen), and the decrease of sex hormone-binding globulin (SHBG) mediated through IR. The GH axis may also be altered in PlwO; GH levels may be lower due to an increase in the GH-binding protein and a GHRH central activation decrease. Serum IGF-1, however, is not altered in PlwO. TSH levels may also be altered due to IR and higher leptin levels (which stimulates TSH secretion). Vitamin D is a fat-soluble vitamin, thus AT vitamin D sequestration may decrease its bioavailability. Renin and aldosterone levels may be elevated through RAS activation in the low-grade inflammation setting [40]. Finally, adiponectin, leptin and ghrelin levels are altered in PlwO [41, 42]. Adiponectin/Leptin ratio (Adpn/Lep) is a suitable indicator of AT dysfunction, thus it may be a useful estimator of cardiometabolic risk [42].

### Renal and genitourinary diseases

PlwO have a higher risk for urinary tract infection [43]. Likewise, obesity and visceral fat are associated with overactive bladder syndrome and urinary incontinence [44]. Moreover, obesity markedly increases the risk of benign prostatic hyperplasia [45]. PlwO may have an increased risk of kidney stones [46]. Furthermore, IR may damage the acid–base kidney metabolism leading to a lower urinary pH together with an elevated uric acid stone disease risk. Besides, refined sugars intake, purine-rich foods, and low fluid intake may contribute to the development of renal lithiasis. Furthermore, Roux-en-Y bypass surgery may in addition augment the kidney stone risk in relation to the elevated hyperoxaluria [47].

Additionally, obesity represents a further risk factor for CKD development [48], even after additional adjustments for blood pressure and T2D. Diabetic kidney disease and obesity-related glomerulopathy are the two main drivers of CKD in PlwO [49]. Obesity-related glomerulopathy, characterized by proteinuria, hypertrophy, and adaptive focal segmental glomerulosclerosis, can subsequently lead to a reduction of the renal function. The hemodynamic, adipose tissue-related, IR common pathophysiology may explain this relationship [50]. VAT, and not subcutaneous adipose tissue (SCAT), assessed by imaging techniques, is associated with a higher albuminuria prevalence [51], suggesting a key role of visceral adiposity in this relation [52]. In this line, studies have also evidenced that prompt identification and management of MAFLD may decrease the CKD burden [53]. However, there is a need of further studies examining the effects of obesity on kidney disease progression.

### Musculoskeletal disease

Obesity can independently lead to loss of muscle mass and function, due to oxidative stress, inflammation and IR [54, 55]. Sedentary lifestyle is both a cause and a consequence of sarcopenia and obesity. Additionally, body fat is associated with widespread and single-site joint pain [56]. Knee osteoarthritis is the most common musculoskeletal comorbidity in PlwO [57]. This comorbid association reduces mobility, which can further increase weight. A recent study showed that living with obesity elevates rheumatoid arthritis risk in women by 40–70% depending on serologic status and age [58]. As expected, weight loss of at least 10% has been associated with an improvement of pain [57]. Moreover, gout, an inflammatory arthritis caused by crystal-deposition subsequent to uric acid serum elevation, is common in PlwO [59]. In all the entities described, weight loss may improve symptoms, nonetheless, gout attacks might occur in the weight loss period [60].

### Neurological diseases

Mounting amount of evidence shows the effects of obesity on the central nervous system [61–63]. In a recent prospective cohort study aiming to clarify the relation between life time adiposity and cognitive impairment, a higher dementia risk was evidenced in people with less fat-free mass and more fat distribution on arms [61]. Neuroimaging studies in PlwO highlight a relation with brain structural abnormalities, mainly temporal and frontal lobes atrophy, corresponding to the executive and memory dysfunctions presented by these patients [62, 63]. A chronic low grade systemic inflammation, oxidative stress, the accumulation of senescence cells in the brain that escalates the neuroinflammation, changes in blood barrier permeability and glial activation have been proposed as responsible for the synaptic remodelling and neuronal apoptosis that has been associated with cognitive impairment in PlwO [64–67]. In PlwO, the etiological implications of vascular pathology in cognitive impairment should not be neglected [68]. Obesity is also connected to idiopathic intracranial hypertension and migraine [69]. At the pathophysiological level, the overlap between migraine and both, central and peripheral pathways, regulating feeding, involving serotonin, adiponectin and leptin is expected [70]. The relation between obesity and peripheral nervous system affects both the somatic nerves causing polyneuropathy [71] and the autonomic nervous system, with an autonomic neuropathy inducing a chronic activation of the sympathetic nervous system [72].

### Psychosocial disorders

One of the most common forms of discrimination in modern societies is weight discrimination [73]. Impressively, negative attitudes about obesity have been evidenced in some healthcare professionals, consequently disturbing patient care [74]. Weight stigma is associated with adverse physiological and psychological outcomes [75]. Obesity stigmatization starts in schools, therefore, children and adolescents living with obesity experience high proportions of bullying and are at an increased risk for social isolation [76]. Later in life, weight-based stigma weakens opportunities for career development and employment. Body dissatisfaction has being identified as a strong correlate with unfavorable obesity-related behavior among PlwO and specially among women [77]. Depression, anxiety disorders, attention deficit hyperactivity disorder, substance abuse, binge-eating, trauma, bipolar disorder, and schizophrenia are the most frequent psychiatric disorders associated with obesity [78].

### Cancer

After smoking obesity accounts for the second cause of cancer that can be prevented [79, 80]. The association of obesity with an elevated cancer risk is observed for esophageal, gastric cardia, gallbladder, colorectal, hepatocellular and pancreatic adenocarcinomas, renal cancer, thyroid cancer, ovarian and endometrium cancer, meningioma, hematological cancer (leukemia, lymphoma, multiple myeloma), prostate, and breast cancer in postmenopausal women [6, 79, 80]. The main pathways linking both entities include hyperinsulinemia, IR, abnormalities of the IGF-1 signaling, low-grade inflammation, oxidative stress, altered intestinal microbiome, and mechanical forces, as elucidated in the common pathophysiology [81, 82].

## Towards a novel diagnostic framework

**Fig. 3:**
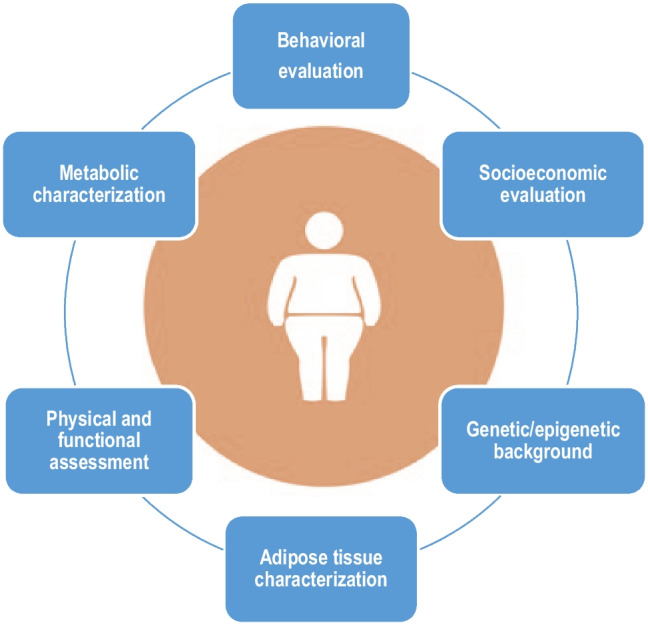
Holistic syndemic approach of people living with obesity Diverse factors influence the phenotype of people living with obesity. Addressing genetics, epigenetics, metabolic, social, lifestyle, and behavioral aspects may help enhancing a better diagnosis and management

Precision medicine allows applying more intensified measures for primary prevention of metabolic abnormalities. Figure 3 summarizes the holistic syndemic approach of PlwO. In the decades to come, it is expected that a broader range of elements that better reflect the complexity of obesity (i.e. genotype, adipotype, microbiome, and exposome) may be evaluated [17, 83].

AT amount and distribution are key features of obesity-related diseases. On the last decade, translational studies have provided evidence that VAT has a strong correlation with metabolic diseases [84, 85]. Therefore, clinicians need to properly evaluate PlwO in a dynamic framework. Anthropometric measures of central adiposity like waist circumference [86–88], WHtR [89–91], and more specifically: VAT [92], VAT to SCAT ratio [93], liver steatosis [94], and epicardial adipose tissue [95], among others, have a central role in the development of impaired metabolic disease. In this line, some normal weight individuals may have excess of VAT and a high cardiovascular risk, exposing the limitations of BMI for health evaluation in the general population [87]. Fatty liver index, abdominal ultrasound or Fibroscan must be performed to rule out MAFLD. Morphofunctional assessment has also shown to provide very useful clinical information. A thorough assessment of all potential obesity-associated alterations should be analyzed in a systematic and holistic way.

**Fig. 4:**
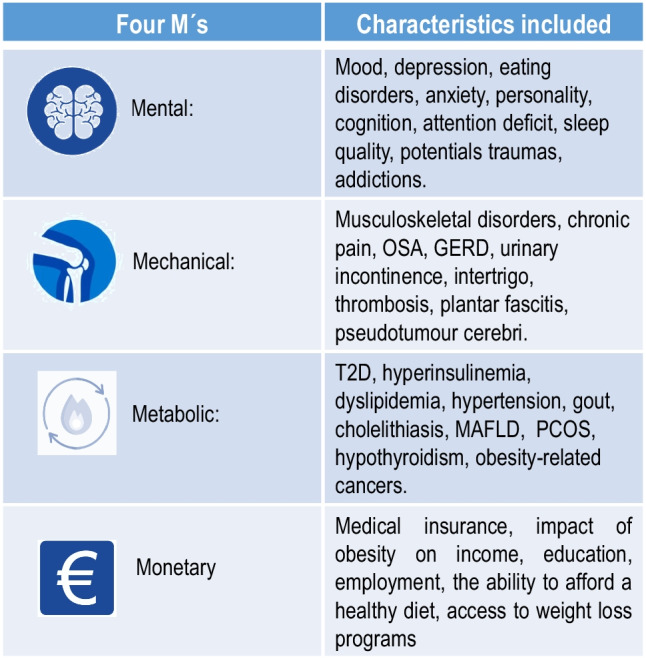
The 4 M’s Mnemonic Framework to assess drivers and complications of obesity [96] Mnemonic framework proposed by the Edmonton Obesity Staging System for the assessment of the patient living with obesity GERD: gastroesophageal reflux disease; MAFLD: metabolic dysfunction-associated fatty liver disease; OSA: obstructive sleep apnea; T2D: Type 2 diabetes mellitus, PCOS: polycystic ovary syndrome

Several aspects may be considered in the precision medicine era for the diagnostic approach of PlwO with a wide perspective, thereby including quite diverse spheres. The Edmonton Obesity Staging System has proposed the use of a mnemonic consisting of four Ms to help the hard-working practitioner navigate through an exhaustive and careful assessment of PlwO [96]. Figure 4 summarizes the mnemonic of the four Ms standing for: mental, mechanical, metabolic and monetary, to assess the drivers and complications of obesity.

Psychosocial evaluation is essential in obesity management with the purpose of identifying potential road blocks and challenges that facilitate behavioral changes aimed at enhancing long-term weight management [73]. Not recognizing mental health issues is likely to result in poor compliance as well as high rates of weight regain [96]. The psychosocial profile is also helpful in identifying potential contraindications for undergoing bariatric surgery (i.e. substance abuse, poorly controlled depression).

Beside the behavioral assessment, socioeconomical evaluation, mechanical, and metabolic comorbidities evaluation through AT dysfunction assessment, serum markers and histopathological features, among others features, should be included in the holistic approach of obesity.

### Inflammation markers

AT synthesizes and releases a number of factors collectively called adipokines, like adiponectin and leptin, closely related to cardiometabolic risk [97–99]. Leptin is predominantly secreted by AT proportionally to AT amount, being directly implicated in food intake control and energy regulation [99]. Adiponectin is known for its anti-inflammatory effect and decreases in PlwO [42]. The Adpn/Lep ratio is reportedly better related with IR than with each of the adipokines alone [98]. In epidemiological studies, an increase in this ratio has been related with a decreased risk of atherosclerosis and some cancer types [100]. An Adpn/Lep ratio ≥ 1.0 can be considered normal, a ratio ≥ 0.5 and < 1.0 indicates moderate to medium increased risk, while a ratio < 0.5 suggests a severe elevation in cardiometabolic risk [42]. Other adipokines [101], like osteopontin, calprotectin [102], pigment-epithelium derived factor [103], ghrelin [104], and adipocyte-derived lipopolysaccharide binding protein [10], are also involved in inflammation and insulin resistance, as is the case with aquaporins [105] and caveolins [106].

In MAFLD, transient elastography [107] and non-invasive markers of fibrosis (e.g. NAFLD Fibrosis Score [NFS] [108] and Fibrosis 4 Score [FIB-4] [109]) have reportedly provided high diagnostic precision in advanced stages of hepatic fibrosis (F3–F4) and associate with MACE [110] and subclinical cardiovascular disease [111]. If available, the enhanced liver fibrosis test (ELF-Test) can be determined as it reflects the liver extracellular matrix metabolism, it measures the levels of amino-terminal propeptide of type III procollagen, tissue inhibitor of metalloproteinases 1, and hyaluronic acid [32, 112].

### Histopathological features

Whenever possible, the histological analysis of AT should be pursued. Histopathological features of AT may predict the possibility of developing diseases associated to obesity or the potential therapeutic response to intervention (i.e. bariatric surgery) [4]. Sampling abdominal subcutaneous and omental AT should be a standard care procedure for PlwO undergoing bariatric surgery. A high fibrosis score in subcutaneous fat [113], a low omental fat mast cell count [114], and a high adipocyte cell size [115], can predict a reduced postoperative weight-loss after bariatric surgery. The balance between lipolysis and lipogenesis is a further relevant aspect given the involvement of adipokines in lipid metabolism regulation and cardiometabolic risk [116–119].

The term “metabolically healthy obesity (MHO)” and “metabolically unhealthy obesity (MUHO)” have been proposed to phenotype and establish risk in PlwO [120]. The MHO definition is still a matter of debate, nonetheless, research has reportedly shown proven risk of CVD not only in MUHO but also in MHO [121–124]. Evaluating subcutaneous adipocyte size in patients with obesity without any comorbid pathology (or “MHO”), may anticipate glycemic control deterioration in patients with even normal glucose tolerance [125, 126], thus metabolic health represents a dynamic marker of elevated risk for progression to unhealthy phenotypes [127]. Inflammatory cytokines concentrations in the diverse obesity phenotypes [5], also support the AT dysfunction continuum gradually leading to the unhealthy phenotype conversion [19, 128].

### Molecular features

In the last decade, studies have identified molecular patterns that could theoretically aid in personalizing obesity care; for example, subcutaneous microRNA expression may be related to the magnitude of weight loss [129, 130]. A higher visceral AT miRNA-122 expression anticipates the magnitude of weight loss following bariatric surgery [131]. Moreover, modern ‘omics’ technologies, single-cell RNA-sequencing of stromovascular fat cells, or single-nucleus RNA-sequencing are potential tools to define specific phenotypes in response to weight loss change based on the underlying complexity of energy homeostasis control and, therefore, may predict response to the diverse therapeutic approaches [4, 17]. In this line, recent studies, have also evidenced that environmental influences affect the epigenetic state, phenotype, and susceptibility to different diseases of next generations [17].

## Addressing innovative therapeutic approaches

In the last years, substantial knowledge related to the biology of obesity has been gained. Unfortunately, comprehension has had little impact on obesity prevalence [84]. The clinical phenotype of PlwO is complex, thereby reflecting the interconnection between environmental, genetic, epigenetic, and lifestyle factors [17]. To appropriately approach the burden of obesity, a paradigm change is needed [83]. Management of obesity requires long-term follow-up to monitor treatment goals, regarding lifestyle changes and comorbidities [132]. Treatment instauration and goals must be personalized based also on the amount and distribution of fat, beyond BMI. Biological, psychosocial, and economic factors influencing health must be considered, individually and globally. Conventionally, approaches are stepwise, lifestyle interventions represent the first step being followed by the application of anti-obesity drugs, endoscopical procedures (e.i. endoscopic gastroplasty, gastric balloon), and consideration of bariatric surgery [32, 133]. However, currently a multimodal approach seems to be better. After bariatric surgery, pharmacological treatment [134, 135] or endoscopic procedures [136] may be further considered for weight regain.

**Fig. 5:**
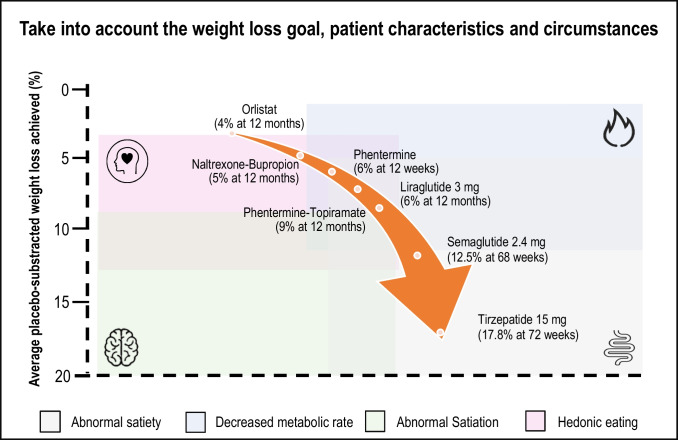
Schematic illustration of plausible phenotype-guided pharmacotherapy Selection of anti-obesity medications centered on energy balance phenotypes (based on Acosta et al) [138]

Patient circumstances, preferences, availability, costs, and comorbidities must be considered in the selection of treatment [137]. Acosta et al. have proposed the ﻿selection of antiobesity medications based on energy balance phenotypes [138]. Interestingly, two or more phenotypes were identified in 27% of PlwO whereas in 15% of the participants, a specific biological phenotype was not identified. Food intake depends on hunger, satiation, gastric emptying, satiety, and emotional eating; and expenditure depends on resting energy expenditure, physical activity, and exercise. In brief, four distinct profiles were identified by these main characteristics: i) hungry brain, ii) emotional hunger, iii) hungry gut and iv) slow brain. Table 1 describes the phenotypes described by Acosta et al. Figure 5 considers energy balance phenotypes and available antiobesity medications. This therapeutic approach has evidenced a more pronounced weight loss as compared to the use of standard care antiobesity pharmacotherapy. Nonetheless, the medication prescribed for each phenotype may be a matter of debate, as GLP-1 receptor agonists may act on different levels, for instance, on abnormal satiation and satiety.

**Table 1:**
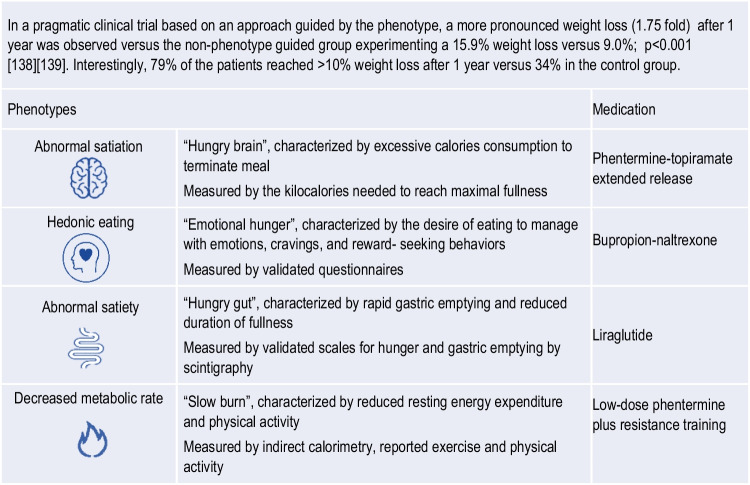
Biological and behavioural phenotype-guided pharmacotherapy to optimize obesity therapy in a precision medicine context

## Conclusions

Obesity is a complex disease affecting almost every organ and system of the body. Clinicians and politicians need to collaborate in the paradigm change characterized by an holistic approach. Future perspectives on adipobiology with innovative novel molecular and histopathological findings may help us predict which patients will respond better to medical, endoscopic, surgical, or mixed treatment. Whilst precision medicine has advanced remarkably in some specialties like oncology, in the field of obesity, progress has been hampered by old-fashioned views of the disease itself, the applied technology for its diagnosis and the scarcity of treatment tools. A long-term comprehensive strategy with multidimensional initiatives focusing on sustainable changes aimed at improving health and well-being rather than achieving a specific weight target should be pursued. Noteworthy, success can be different for every individual ranging from a better quality of life to greater self-esteem, a 5% weight loss, a decrease in cardiometabolic risk factors, prevention of weight regain, among others.

## Abbreviations

ABCD: Adiposity-based chronic disease
Adpn/Lep: Adiponectin/leptin ratio
AT: Adipose tissue
CKD: Chronic kidney disease
CVD: Cardiovascular disease
ELF-Test: Enhanced liver fibrosis test
FFA: Free fatty acids
GERD: Gastroesophageal reflux disease
IR: Insulin resistance
MACE: Major adverse cardiovascular events
MHO: Metabolically healthy obesity
MUHO: Metabolically unhealthy obesity
MAFLD: Metabolic associated fatty liver disease
OHS: Obesity hypoventilation syndrome
OrAD: Obesity-related adipose tissue disease
OSA: Obstructive sleep apnea
PlwO: People living with obesity
RAS: Renin-angiotensin-aldosterone system
SCAT: Subcutaneous adipose tissue
SHBG: Sex hormone-binding globulin
T2D: Type 2 diabetes
VAT: Visceral adipose tissue
WHtR: Waist to height ratio
